# Relational Integration and Attentional Control Are Crucial to Fluid Intelligence Together but Not Alone—An Experimental Investigation of Individual Difference in Relational Monitoring Processes

**DOI:** 10.3390/jintelligence14010008

**Published:** 2026-01-05

**Authors:** Yunze Li, Damian Patrick Birney

**Affiliations:** School of Psychology, University of Sydney, Camperdown, NSW 2006, Australia; yunze.li@sydney.edu.au

**Keywords:** fluid intelligence, relational integration, attentional control, working memory, relation monitoring task

## Abstract

Working memory (WM) and fluid intelligence (Gf) are highly correlated, which provides the basis for the claim that they share common cognitive processes. Attentional Control Theory and the Relational Integration Hypothesis are two process theories linking WM and Gf. Additionally, both have empirical evidence to support them; the strength of this evidence can be limited by the experimental manipulations used and the operationalisation of performance metrics. To investigate the cognitive processes related to Gf, levels of relational integration and attentional control in the relation monitoring task (RMT) were manipulated. Study 1 (N = 39) focused on calibrating RMT response time windows for different levels of relational integration to strengthen validity claims by reducing possible ceiling effects in RMT performance observed in prior research. Study 2 (N = 146) examined how Gf was related to manipulations of relational integration and attentional control. The research extends previous studies by (a) using experimental manipulations that align more closely to underlying process accounts, and (b) contrasting simple-composite scores, a common operationalisation of performance, with a variance decomposition approach that statistically isolates the hypothetical processes aligned with the experimental manipulations. Results suggest that the way performance is operationalised matters, and that neither relational integration nor attentional control processes alone relate to Gf; instead, predictive utility is greatest when they are operationalised together.

## 1. Introduction

Gf is an extensively studied construct; surprisingly, its underlying cognitive processes remain disputed, with many questions remaining. Over recent decades, numerous attempts have been made to understand such processes by studying the link between Gf and working memory (WM) in order to explain the ~50–70% of common variance (Bateman, 2020; Kovacs & Conway, 2016; Oberauer et al., 2005). Attentional Control Theory of WM and the Relational Integration Hypothesis are two influential and distinct theories that attempt to explain the WM–Gf relationship. However, a clear consensus on even how to achieve this has yet to be reached (see, for instance, Frischkorn & Oberauer, 2021).

Attentional Control Theory, stemming from the work of Engle and collaborators in the late 1990s (e.g., Engle et al., 1995), proposes that executive/controlled attention is the common process underlying both WM and Gf (Shipstead et al., 2016; Unsworth & Engle, 2005, 2007). More recent work from this perspective suggests there are two core sets of processes under the coordination of executive attention: maintenance and disengagement. The proposal is that maintenance is more critical to WM tasks, whereas Gf tasks demand more disengagement (Shipstead et al., 2016). Although the attentional control explanation of the WM–Gf relationship has received considerable empirical support (e.g., Draheim et al., 2021; Kane et al., 2005), many other studies indicate otherwise (e.g., Oberauer et al., 2008). For instance, Rey-Mermet et al. (2019) suggested that the measurement of executive attention is confounded with processing speed and that executive attention itself does not relate to Gf. Mashburn et al. (2021) provided an alternative explanation that most executive attention tasks, including those used by Rey-Mermet et al. (2019), suffer from low reliability because the dependent variable is a difference score between task conditions, which is the reason for the low correlations (see also Draheim et al., 2021).

**Figure 1:**
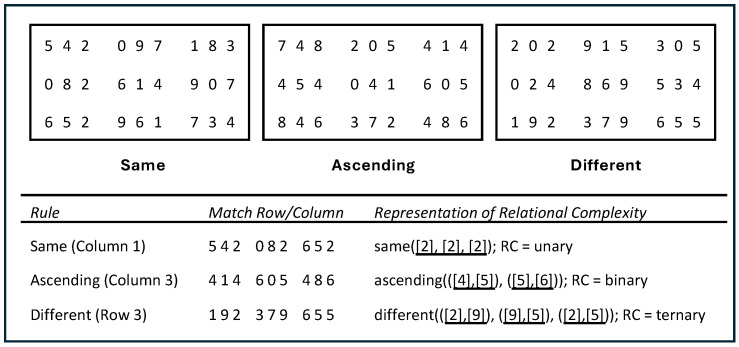
Example RMT items and relational complexity analysis for the same, ascending, and different trials. Note: RC represents relational complexity.

From a relational integration perspective, the essential common process shared by WM and Gf is relational integration (Bateman, 2020; Oberauer, 2021). It involves the binding between contents (e.g., objects, people, events) and contexts (e.g., roles, schemas, spatial coordinates in mind), which are then integrated within the focus of attention to form structural representations (Oberauer, 2009). To capture relational integration, Oberauer et al. (2003) developed the relation monitoring task (RMT), which requires participants to monitor a periodically changing matrix. In one variant of the task, the matrix contains 3 × 3 three-number strings and participants need to identify matches based on predetermined match rules (Figure 1). The task is theorised to require relational integration because the target elements (the last digit of the string in a row or column) need to be bound to spatial coordinates in WM and integrated into a relation for testing against the match rule. Empirically, a latent relational integration factor extracted from varied versions of RMT can well predict Gf to a similar level and beyond traditional WM tasks (e.g., operation span and n-back task; Bateman et al., 2019; Chuderski, 2014; Oberauer et al., 2008).

In this paper, we aim to clarify two key issues. First, we examine the distinctions between the process accounts proposed by Attentional Control Theory and the Relational Integration Hypothesis, and how each relates to Gf. Second, we contrast a common methodological approach based on simple composite scores with a variance–decomposition approach. The variance–decomposition approach, similar to that used by Ecker et al. (2010) employing SEM, begins by operationalising hypothetical cognitive processes with explicit task manipulations that serve as within-task conditions. These task conditions are then formalised statistically as latent variables using multilevel modelling.

### 1.1. Relational Integration in RMT

The relational integration demand in RMT can be understood according to relational complexity theory proposed by Halford et al. (1998, 2010). Specifically, the theory can be conceptualised in RMT using the three match rules: same, ascending, and different (Figure 1), which requires increasing relational integration demand from unary to ternary^1^ (Bateman et al., 2019). Verification of a “same” match rule represents unary integration, because the three numbers can be chunked together into one relation. An “ascending” match requires binary integration, where (in Figure 1, *Ascending*) the targets [4,5,6] are chunked into two relations ASC [4,5] and ASC [5,6]. Integrating these two relations leads to the implication that the whole string [4,5,6] entails an ascending relation. A “different” match requires ternary integration. If one wants to determine that [2,9,5] are three different numbers (in Figure 1, *Different*), one must know that the elements of the independent sets [2,9], [9,5], and [2,5] are different. Integration of these three separate relations is needed to verify a “different” match; no chunking is possible here. Empirically, consistent with relational complexity theory, RMT accuracy decreased when relational complexity increased, and its relationship with Gf became stronger (Bateman et al., 2019; Chuderski, 2014). Together, these studies reinforced the Oberauer et al. (2008) argument that relational integration is an essential function of WM and Gf, which is better captured in RMT than traditional WM measures.

### 1.2. Attentional Control in RMT

Given the criticism of unreliable attentional control measures and the potential confounding of constructs, the processes underlying the Relational Integration Hypothesis have received more consistent evidence as candidate processes common to Gf and WM. However, these two process accounts are not necessarily mutually exclusive. Although the Relational Integration Hypothesis heavily focuses on the need to form relational structures in WM, integration inevitably involves attention (Oberauer, 2009; Oberauer & Lewandowsky, 2016). This raises two important questions: (1) To what extent does relational integration involve attentional control? And (2) Which processes are most closely related to Gf? To answer both questions, teasing apart attentional control from relational integration is crucial.

**Figure 2:**
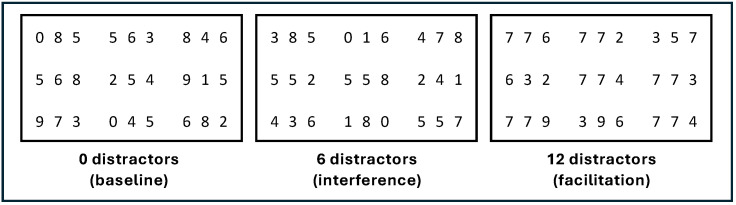
RMT Items with 0, 6 and 12 Distractors. Note: The distractors always come in pairs and are located at the first two digits of a string. Number “5” is the distractor in the interference condition. Number “7” is the distractor in the facilitation condition. All three examples are non-matched items (the match rule is “same”). This figure is adapted from Zhan and Birney (2023).

Moreover, RMT was initially designed to measure relational integration; previous studies also claimed that it involves two related but distinct attentional control processes: *visual interference* (i.e., inhibition) and *rapid scanning of strings* (Bateman et al., 2019; Chuderski, 2014; Krumm et al., 2009). This provides an opportunity to tease apart attentional control from relational integration by manipulating one demand while controlling for the other in RMT. When selecting the target end digits from the matrix, the first two digits can be considered as distractors (Zhan & Birney, 2023). Under the coordination of attentional control, it is important to maintain attention on the target stimuli by *inhibiting* the non-target digit distractors and then reengaging with the task if attention is distracted by disengaging with unrelated task information. Following Bateman et al. (2019) and Chuderski (2014), Zhan and Birney (2023) introduced three levels of visual interference: 0, 6, and 12 distractors at non-target digits (Figure 2). They discovered that having six distractors at non-target digits reduced RMT performance, while having twelve distractors negated this detrimental effect, and instead facilitated performance, presumably by making the end-digits more salient. However, the impact on RMT performance was only observed in the “different” condition. Their findings may suffer from a ceiling effect because the response time limit for each RMT trial may be too long (5.0 s), especially for lower relational complexity blocks. Thus, their result may be biassed toward a smaller effect of attentional control on RMT performance. This is addressed as a core aim of our Study 1.

**Figure 3:**
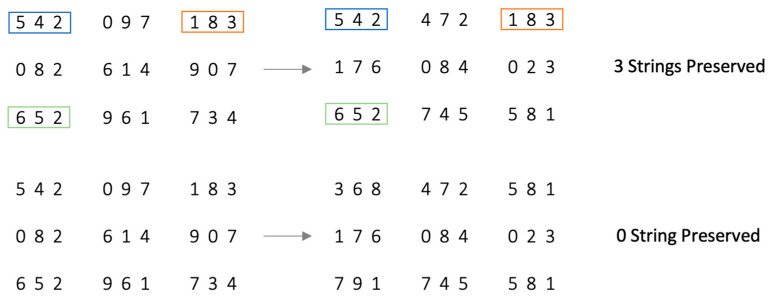
Illustration of string preservation. Note: From trial 1 to 2, the top image illustrates that 3 strings are preserved (6 strings are replaced), and the bottom image illustrates that 0 strings are preserved (all 9 strings are replaced).

Further, the limited display time of the RMT also means that a successful solution requires rapid scanning of the nine end-digits in the matrix. As the number of strings scanned increases from trial to trial across the block, outdated strings from previous trials need to be disengaged to allow strings from the current trial to be encoded and maintained for consideration (Figure 3). Bateman et al. (2019) introduced two conditions that manipulate this specific attentional control demand related to scanning: In the first condition, 1 to 4 strings were randomly carried across (i.e., preserved) from the immediately preceding trial to the current one. In the second condition, all strings from the preceding trial were replaced (i.e., the current trial contained no strings from the previous trial). The expectation was that, relative to replacing all strings, preserving some strings from the previous trial would reduce scanning demand, leading to better performance. Although Bateman et al. (2019) found no mean differences in RMT performance between the two conditions, regression analyses suggested performance in the string-replaced condition (greater attentional control demand for scanning) incrementally predicted Gf over and above the string-preserved condition (less attentional control demand). Together, this suggests partial support for disengagement/encoding as an important aspect of Gf. The lack of a statistically significant mean differences between conditions in their study may be attributed to the small structural difference they implemented—almost half of the trials designated as string-preserved had little actual difference from the all string-replaced trials (i.e., trials in which only one or two strings were preserved), and it may have been this that contributed to an underestimation of the true attentional control effect. In the current study, the experimental manipulation was made more distinct by having three clearly defined levels: zero (all replaced), three, and six preserved strings.

### 1.3. Analytical Approaches: Operationalising Performance Metrics to Test Processes

Simple-Composite Scores: The experimental studies reported above used simple-composite scores of the various task conditions and sequentially entered these into a standard regression to test for a change in *R*^2^. For example, consider a 2 (low vs. high relational complexity) × 2 (low vs. high visual interference) design. The complexity effect would be tested with two composite variables derived by aggregating across all interference trials separately for the low- and high-complexity trials. Results showing that the high-complexity variable provided incremental prediction of Gf over and above the low-complexity variable would be interpreted as evidence that complexity is an important process underlying Gf. Furthermore, since no difference score is required in computing the variables, the low reliability issue of classic attentional control measures can be bypassed to some extent. However, this statistical approach raises a validity issue since the simple-composite score overlooks the dependency between the derived task variables. When adding a high relational complexity score into a regression model already including a low relational complexity score, the increase in explained variance is assumed to be due to the heightened relational integration demand. However, it may also arise from the demand for visual interference, especially if the underlying interference effects vary based on the level of relational complexity (e.g., homoscedasticity of interference effects does not hold at all relational complexity levels). Therefore, both Bateman et al. (2019) and Zhan and Birney’s (2023) results, indeed any that use this methodology, may need to be interpreted with some caution.

**Figure 4:**
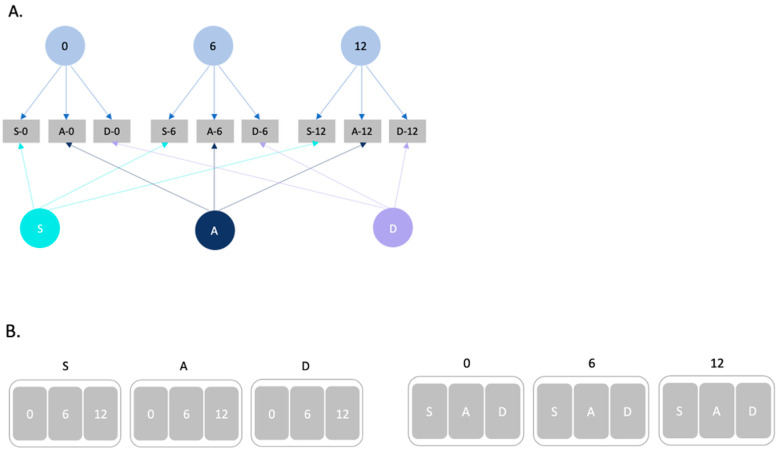
Difference between (**A**) latent contrast scores and (**B**) simple-composite scores. Note: Part (**A**) illustrates how variance-decomposition processes from a hypothetical 3 (relational complexity: same (S), ascending (A), different (D)) × 3 (number of distractors: 0, 6, 12) within-subjects design. Each circle represents a latent variable, and each square represents the observed RMT item performance in different conditions. For example, S-0 represent trials of the “same” condition that have no distractor. Part (**B**) illustrates how simple-composites scores are derived from the same set of hypothetical data. The derived scores of each relational complexity level contain the variance of each distractor level (**left**). The derived score of each distractor level contains the variance of each relational complexity level (**right**).

Latent Contrast Scores: To achieve our analytic aim, in the current study, we contrast the simple-composite score approach described above with a variance-decomposition approach using multi-level modelling (MLM). With MLM, latent variables can be defined for each experimental manipulation (Figure 4) where trial-level accuracy scores (0/1) are modelled as the random-effect of the experimental condition the trial represents (across repeated trials), clustered within individuals. For example, when comparing the 0-distractor and 6-distractor conditions, a variable contrasting the “0” and “6” levels of the distractor condition is derived as an effect-contrast. This contrast variable represents the cost of interference, operationalised as a comparison between the individual’s performance on trials that have six non-ending distractor digits relative to trials that have no repeated non-ending distractor digits (see Figure 2). Including relational complexity levels in the analyses statistically controls for complexity effects that are problematic in the simple-composite approach. At its core, this is a variance-decomposition approach, where the effects of experimental manipulations are contrasted as (latent) random-effect variables in the MLM. This is argued to better address the low reliability of attentional control measures (as difference scores, for instance) without incurring the validity issue often left untested in the simple-composite approach (Birney & Beckmann, 2022). The critical test can then be conceptualised as the extent to which the effect of the latent-contrast score on RMPT performance is moderated by Gf. This is analogous to the one-step approach described by Brose et al. (2021), albeit using an MLM regression approach rather than multi-level SEM or other SEM modelling frameworks (e.g., Ecker et al., 2010).

### 1.4. The Current Study

The current study manipulated the level of relational integration (relational complexity demand) and attentional control (interference and visual-scanning demands) in the RMT to investigate how performance is impacted by these different theoretical processes and the extent to which each is associated with Gf.

Improvements were made to better optimise the experimental power to distinguish attentional control from relational integration. We reconstructed RMT items based on Bateman et al. (2019) and Zhan and Birney (2023) to enhance the manipulation of string preservation by utilising a wider range across trials. Meanwhile, to determine the appropriate response windows for each relational complexity level, in Study 1, we analysed the variances of RMT performance elicited by using different response time criteria. Then, in Study 2, we applied the response time suggested by Study 1 with the revised RMT.

### 1.5. Hypothesis

#### 1.5.1. Relational Integration (Relational Complexity)

After controlling for attentional control, as relational complexity increases, RMT performance decreases (H1), and if this is a process common to Gf, then Gf is expected to moderate the cost of increasing relational complexity (H2).

#### 1.5.2. Attentional Control-Inhibition (Visual Interference)

After controlling for relational integration, having six non-target distractors to be inhibited is predicted to produce poorer RMT performance than having 0 and 12 distractors (H3). Further, having 12 distractors is predicted to yield better RMT performance than having no distractors (H4), as it serves to narrow the focus of attention away from irrelevant stimuli. According to Attentional Control Theory, Gf entails organising executive attention to inhibit irrelevant distractors. Therefore, Gf is predicted to moderate the cost of increasing visual interference demands, after controlling for relational integration (H5).

#### 1.5.3. Attentional Control-Scanning (String Preservation)

After controlling for relational integration, having more strings preserved is predicted to lead to better RMT performance than having fewer strings preserved (H6). This is because fewer stimuli need to be scanned and encoded when strings are preserved across trials. If this is a process common to Gf, then Gf is predicted to moderate the cost of increasing visual-scanning demands, after controlling for relational integration (H7).

## 2. Study 1

### 2.1. Overview

This pilot study aimed to determine an appropriate response window for different levels of relational complexity to avoid the potential ceiling effects observed in previous studies that may have impacted tests of the process theories of interest. To achieve this, Zhan and Birney’s (2023) data were re-analysed to derive prospective shortened response windows. Since the ceiling effect appears as reduced response variance in data, we argued that all else equal (and up to a point, e.g., Pedhazuer & Schmelkin, 1991), the condition with higher RMT performance variance was more likely to mitigate potential ceiling effects. In Zhan and Birney (2023), a ceiling effect was not apparent for the “different” condition, which suggested that this condition may be difficult enough to differentiate participants’ performance. As it is not known whether participants would approach lower complexity RMT trials differently under reduced response windows (e.g., invest more effort, or use different scanning strategies), we ran a preliminary parameter-setting study to investigate this. Our goal was simply to identify a response window that resulted in a statistical variance sufficient to mitigate a performance ceiling.

### 2.2. Method

#### 2.2.1. Three Potential Criteria to Reduce Response Windows

We calculated the timeout rate of RMT trials in Zhan and Birney (2023), defined as the average proportion of trials that participants failed to respond to within the set 5 s response window. On average, the “different” condition had a timeout rate of 10%, whereas the “same” condition had 2.8%, and the “ascending” condition had 7%. Assuming the 10% timeout rate represents a sufficient level of difficulty (in other words, anchoring difficulty based on the “different” condition), setting the trial response window to correspond to a 10% timeout rate for the “same” and “ascending” conditions may also successfully address the ceiling effect. As a contrast, we also calculated the upper quartile (75th percentile) and the median (50th percentile) RMT response time as two additional sets of response windows to investigate. Table 1 lists the RMT response windows derived from Zhan and Birney (2023) for each relational complexity level to be used in Study 1.

#### 2.2.2. Relational Monitoring Task

The version of RMT in Study 1 was adapted from Bateman et al. (2019) and Zhan and Birney (2023). Each trial of RMT involved a 3 × 3 matrix. Each matrix–cell contained a three-digit string. All digits were numbers from 0 to 9, and the content of the RMT updated from trial to trial. For each matrix–cell, only the last digit was a potential target to be matched with a predetermined rule. For the current study, there were only three rules: same, ascending, and different.

To prevent any possible confusion regarding the matching criteria, the “different” condition deliberately excluded strings that could be misconstrued as matches in either the “same” or “ascending” conditions. For example, the ending digits such as [1,1,1] (same) or [1,2,3] (ascending) never appeared in the “different” condition. Similarly, ending digits that qualify as a “same” match never appeared in the “ascending” condition and vice versa.

Participants pressed “L” on the keyboard when they observed a match and “A” when they could not find a match. After reading the instructions, participants underwent six training trials for each matching rule, with correct/incorrect feedback provided. In the formal task, the current match rule was always displayed centred at the top of the screen, and a response reminder (A = no match; L = match) was displayed at the bottom of the screen. The matrix appeared at the centre of the screen. The interval between trials was fixed at 0.1 s, and no feedback was provided for the formal task. There were two RMT components—in addition to relational integration, one tested the inhibition hypothesis, and the other tested the scanning hypothesis.

#### 2.2.3. RMT Component 1 (Relational Integration and Inhibition)

**Figure 5:**
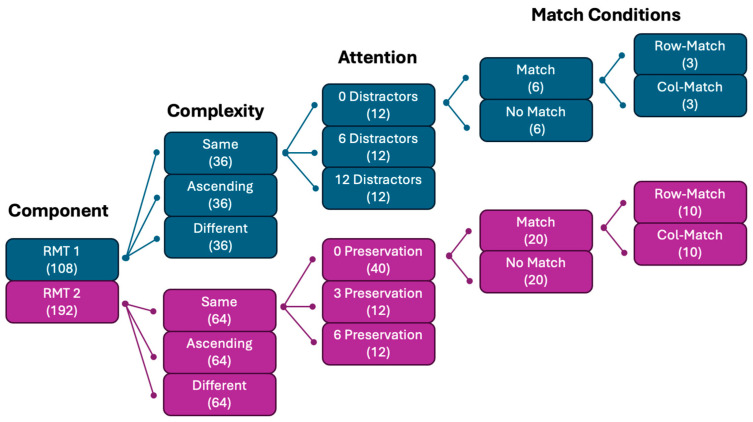
Trial design of RMT Components. Note: The number in brackets refers to the number of RMT trials. “Ascending” and “Different” blocks have the same structure as the “Same” block.

In RMT Component 1, there were three blocks of items with different relational complexity levels (see Figure 5). Each block contained 36 trials, and the visual interference levels were balanced across each block (12 trials for each visual interference level). These visual interference levels were distributed in random order within a block. The distractors would not appear in all non-ending positions within the same row or column (e.g., 556, 558, 550 would never appear in any row or column), because they could excessively direct participants’ attention to the final digits and potentially disrupt interference effects.

Further, within each block, half of the 36 trials were match trials, and the other half were non-match trials. Within the match trials, half of the trials involved row-match, and the other half involved column-match. Controlling the amount of row-match and column-match trials is important, as Zhan and Birney (2023) demonstrated that, on average, row-match trials are more difficult than column-match trials. Meanwhile, the selection of the distractor number on one trial depends on whether it was a match trial or a non-match trial. In match trials, one of the three ending digits involved in the match was the distractor. In non-match trials, a random ending digit was selected as the distractor. The RMT items for this component were directly sourced from Zhan and Birney (2023).

#### 2.2.4. RMT Component 2 (Relational Integration and Visual Scanning)

In RMT Component 2, participants completed three blocks with different relational complexity levels (see Figure 5). Each block contained 64 trials. Within each block, the first trial was a 0-preservation trial, and the second trial randomly preserved 0, 3, or 6 strings from the previous trial; the rest of the trials followed the same logic. If the previous trial had strings preserved, then the subsequent trial must be a 0-preservation trial. The reason for having this structure was to eliminate the possibility of any strings being preserved across more than one trial. As a side effect, RMT Component 2 had an imbalanced number of items in different string preservation conditions. Thus, in addition to the randomness, the study required each participant to complete 12 three-string-preserved trials, 12 six-string-preserved trials, and 40 zero-string-preserved trials. These RMT items were generated using RStudio (version 2025.09.1+401; R Core Team, 2025) with the package ‘tidyverse’ (Wickham et al., 2019) and ‘sets’ (Meyer & Hornik, 2023). In total, four sets of items were produced for each relational complexity level, and participants were randomly assigned to one of four versions of the task. This step was essential to minimise potential order effects present in the task. All other elements were the same as Component 1.

### 2.3. Participants and Procedure

Thirty-nine participants sourced from Prolific (www.prolific.com) provided informed consent and participated in Study 1. Participants were randomly allocated into one of the three response-window conditions, which used the upper quartile, median response time, and response time fixed at the 10% timeout rate from Zhan and Birney (2023) as the response window for RMT. In total, there were six blocks of trials (three for each component), and the order of these blocks was counterbalanced among participants. After that, participants filled out demographic information, including age, gender, and English competency, before receiving the debrief. The whole experiment was administered using Inquisit 6 (www.millisecond.com) and took approximately 30 min.

### 2.4. Results and Discussion

Four participants were excluded due to non-serious attempts based on the following criteria: (1) an unreasonably quick RMT trial response time, defined as the upper quartile of the participant’s response time on RMT trials < 0.75 s and the lower quartile of the participant’s response time < 0.25 s; (2) a mean accuracy for any RMT component was lower than the 50% chance level. The data from 35 participants (18 male, 20 female, *M*_age_ = 35.87, *SD*_age_ = 14.07) were analysed.

**Figure 6:**
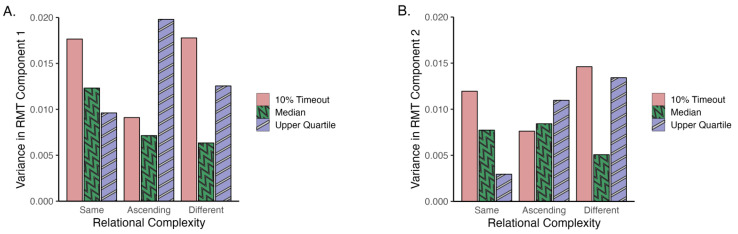
Variance in RMT performance for each response window condition in (**A**) component 1-inhibition and (**B**) component 2-scanning.

Figure 6 illustrates the result of Study 1. Our goal was to identify a response window for each relational complexity level with the largest raw performance variance; no statistical testing was conducted. Within each relational complexity level, both RMT Components 1 and 2 provided consistent results. Specifically, using the 10% timeout rate as the criterion for RMT induced the largest amount of variance in both “same” and “different” conditions for both Component 1 and 2. Meanwhile, using the upper quartile as the criterion for the RMT induced the largest amount of variance in the “ascending” condition for both components. Given that the ceiling effect appears as reduced response variance in data, the condition with higher RMT performance variance was more likely to alleviate the ceiling effect. Therefore, Study 1 suggested that setting the RMT response window as 4.05 s for the “same” condition (response window at the 10% timeout rate), 4.41 s for the “ascending” condition (upper quartile response window), and 5.0 s for the “different” condition (response window at 10% timeout rate) may help alleviate the potential ceiling effect found in previous studies.

## 3. Study 2

### 3.1. Experimental Design

The design of Study 2 involves manipulating three independent variables, relational complexity (relational integration), visual interference (inhibition), and string preservation (scanning), each with three levels. The relational complexity manipulation involves the same, ascending, and different match rules. The visual interference manipulation involves 0, 6, and 12 repeated non-target digit distractors (where 0 indicates no distractor). Finally, the string preservation manipulation involves 0, 3, and 6 strings preserved from the previous trial to the current one. Here, 0 means no strings are preserved, which in effect defines a complete replacement trial.

It is important to note that a fully crossed 3 × 3 × 3 within-subject design complicates and potentially confounds the manipulations, thus risking a weakening of the main effects. Consider an example where the previous trial contains 12 distractors. If the next item is from the condition where 6 strings are preserved from that previous trial, then it would be impossible to have 0 distractors. Further, the distractor number will very likely remain unchanged from the previous trial to the next trial if the next trial has 3 or 6 distractors. Due to this, the current study spread the manipulations across two 3 × 3 within-subject components, as described in Section 2.2.3 and Section 2.2.4. Two 3 × 3 designs are appropriate, as we are not interested in the interaction between string preservation and visual interference because theoretically, they fall onto the same attentional control construct.

### 3.2. Method

#### 3.2.1. Sample

A G*power analysis (Faul et al., 2009) was conducted based on an *R*^2^ increase of *η*^2^ = .08 in a linear multiple regression. This was estimated from considerations of previous RMT studies across different experimental manipulations (e.g., Bateman et al., 2019; Chuderski, 2014; Zhan & Birney, 2023). The number of tested variables was set at 3 (condition-effect, Gf, and conditionxGf), with the total number of predictors set at 5 (i.e., with the addition of covariate control variables). With power set to .80 and type I error controlled at .05, as is conventional, the estimated sample size required is 130.

One hundred and seventy-seven first- and second-year undergraduate psychology students from the University of Sydney who received course credit for participation in the study. Thirty-one participants were excluded for the following reasons: (1) an unreasonably quick RMT trial response time, defined as the upper quartile of participant’s response time on RMT trials < 0.75 s and the lower quartile of participant’s response time < 0.25 s (n = 5); (2) a mean accuracy for any RMT component was lower than the 50% chance level (n = 3); (3) Attention check questions in Gf tasks were not passed (n = 6); (4) a non-serious attempt evidenced by a score of 0 on any of the Gf tasks (n = 5); and (5) a technical error (n = 12). Finally, data from 146 participants (36 male, 109 female, *M*_age_ = 19.39, *SD*_age_ = 1.53) were analysed. 

#### 3.2.2. Relational Monitoring Task

The RMT used in Study 2 has the same block and trial design and interface as the one used in Study 1. However, the response window for each trial differed depending on the match rule. The decision is made based on the Study 1 results, which were “same” (4.05 s), “ascending” (4.41 s), and “different” (5.00 s).

#### 3.2.3. Gf Measures

Three Gf measures were used to form a latent Gf variable as a covariate/moderator. These three tasks are adapted from Gf measures in Bateman et al. (2019).

Raven’s Advanced Progressive Matrices (RPM): Compared to Bateman et al. (2019), which utilised a 20-item version, due to the limitation of restricted testing hours, the current version only consisted of 12 items. Every third item was selected from Raven’s (1989) original 36-item test and presented sequentially, starting at item 1. Participants were given 10 min and instructed to accurately solve as many questions as possible. Items not completed within the time limit were scored as incorrect. The maximum achievable score was 12.

Number Series: The study gave participants 4 min to solve as many number series items as they could. In total, there were 15 items presented sequentially in order of difficulty. Number series is another classic Gf test and usually shares factor loadings with the latent Gf factor above .70 (Kvist & Gustafsson, 2008; Draheim et al., 2021). Participants need to determine the number that comes next in the number series and enter this into a free-field text box. The maximum achievable score is 15.

Greco-Latin Square Task (GLST): GLST items involve a 5 × 5 matrix in the design of two superimposed Latin squares, and it was validated as a measure of Gf by Birney et al. (2012). Participants were informed that one shape could only appear once for each row and column. The same rule also applies to colours. Each combination of shape and colour in the matrix was also unique. Participants were required to determine the shape and colour of the target cell. They were given 10 min to solve as many of the 12 as possible accurately. Items were presented sequentially in increasing order of difficulty. For scoring, an item is considered correct only if both colour and shape are correctly answered. The maximum achievable score is 12. 

#### 3.2.4. Fluid Intelligence

The Gf factor was extracted using exploratory factor analysis (using R package ‘psych’ (version 2.5.6) with promax rotation and maximum likelihood extraction) based on the three Gf measures—RPM (Cronbach α = .60), number series (Cronbach α = .74), and GLST (Cronbach α = .56). The Gf factor accounted for 24% of the variance in these three measures. The factor loading was .47 for RPM, .49 for number series, and .52 for GLST. The internal consistency (α) for RPM and GLST in our study is low, which is likely due to the reduced number of items (compared to Bateman et al., 2019), given the limited testing time. Meanwhile, when all three Gf measures were combined, the reliability is at an acceptable level (Cronbach α = .75).

#### 3.2.5. Procedure

After informed consent was obtained, participants completed the RMT first. In total, there were six blocks of trials (three for each component), and the order of these blocks was counterbalanced among participants. Then, participants completed three Gf tasks. The order of these Gf tasks was also counterbalanced. After that, participants filled in demographic information, including age, gender, and their English competency, before they received the debrief. Participants completed the study in the lab in groups no bigger than 10. The entire experiment was administered using Inquisit Version 6 (www.millisecond.com) and Qualtrics Version XM (www.qualtrics.com), and it took approximately 1 h.

### 3.3. Results

**Figure 7:**
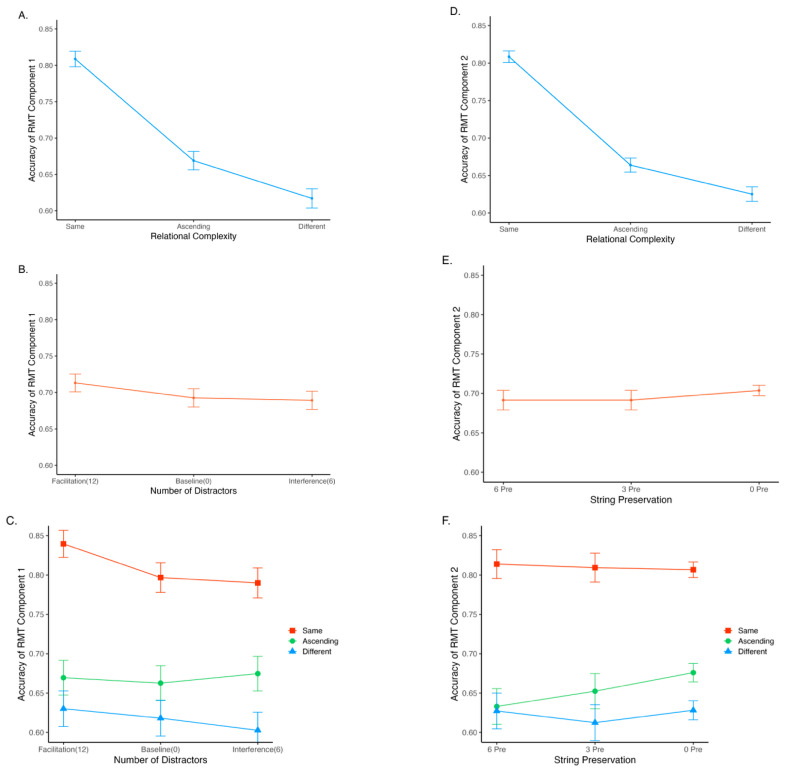
Summarised raw data of RMT component 1 (**A**–**C**) and component 2 (**D**–**F**). Note: The error bars represent two standard errors. “Pre” represents preservation.

The data analysis involved 146 participants with a total of 44,676 observations after implementing the exclusion criteria (15,768 for RMT component 1; 28,908 for RMT component 2). The data were analysed using RStudio version 4.2.2 (R Core Team, 2025). Specifically, RMT performance was analysed with MLM, and both MLM and ordinary least squares (OLS) regression methods were used to explore the relationship between different RMT conditions and Gf. Packages ‘tidyverse’ (Wickham et al., 2019), ‘readxl’ (Wickham & Bryan, 2023), and ‘stringr’ (Wickham, 2022) facilitated data cleaning and processing. For statistical analysis, ‘psych’ (Revelle, 2023) and ‘lme4’ (Bates et al., 2015) were utilised, while ‘sjPlot’ (Lüdecke, 2025) and ‘ggplot2’ (Wickham, 2016) were employed for graph generation. Table 2 displays the descriptive statistics and correlations, and Figure 7 offers a summary of the raw data.

The hypotheses were tested by implementing a multi-level logistic regression from the ‘lme4’ package using the ‘glmer’ function (Bates et al., 2015). The correctness of each item (a binary output: 0/1) was predicted by the contrasts developed from our manipulations and hypotheses (see Table 3). For example, the predicting contrast, which tests “same” versus “different” conditions, represents a predicted performance cost from “same” to “different” (an increase in relational complexity demand). In terms of the predicted variable (correctness), this cost manifested as a decrease in the log-odds chance of answering the item correctly. To link the RMT performance with Gf, Gf was included in the model as an interaction term with each contrast. In other words, this is testing whether Gf can moderate the costs from each contrast. For example, Gf may moderate the “same” versus “different” contrast in a way that people with lower Gf incur greater cost for them when relational integration demand increases, whereas people with higher Gf incur less cost. This result from MLM (the variance decomposition approach) was then juxtaposed with the simple composite results from OLS regression.

#### 3.3.1. RMT Contrast Variables

Hypotheses were tested by deriving contrast variables for each experimental manipulation. To maintain independence, specific contrasts were chosen to be orthogonal within their condition. We describe these contrasts below. Coding values are provided in Table 3.

Relational Integration Cost

The “same”, “ascending”, and “different” conditions are the operationalisation of an increase in relational integration demand (as indexed by relational complexity theory). For the MLM analyses, this was coded as linear and quadratic trends.

Attentional Control-Inhibition

The three levels of inhibition demand (distraction) were operationalised as the number of non-ending digits repeated. Two contrasts were of interest. First, the high distraction cost (HighD), which contrasted the 12 and 0 distractor conditions (low) with the 6 distractor condition (high). The second contrast compared the two low distraction conditions (LowD), i.e., 12 distractors vs. 0 distractors.

Attentional Control-Scanning

The three levels of the attentional demand condition were characterised by whether zero, three, or six strings were pseudo-randomly chosen to persist across from the previous trial. The first contrast tests the preserved cost (PreserveC), i.e., strings-preserved conditions (low) vs. no strings preserved condition (high). The second contrast compared the preserved level conditions (PreserveL: the six vs. three string preserved conditions).

#### 3.3.2. Latent Construct Scores

RMT Performance Effects

The first MLM model tested how relational complexity and visual interference impact RMT performance (Table 3) and whether visual interference moderated the relational complexity effect. It was planned to include random effects for all contrasts except the quadratic control contrast of relational complexity, which was entered as a control variable due to a potential quadratic pattern observed in the data (see Figure 7A). However, fitting the model caused a near-singularity issue because the variance of the random effects of the contrasts “HighD” and “LowD” was very close to zero. Here, we report the model that estimates the random effect for “RC” and the intercept only.

It is prudent to reflect on the implications of various comparisons made in terms of power and alpha inflation. We note, as have others (Birney et al., 2017), that the MLM approach is distinctly advantageous in this regard (Gelman et al., 2012) compared to OLS regression (Brunner & Austin, 2009). MLM uses a partial-pooling process (often referred to as “shrinkage”) that serves to shift parameter estimates and their associated standard errors toward the mean coefficient in the complete data. This process has the desirable effect of shrinking coefficients that are estimated with small accuracy more so than those estimated with higher accuracy (Hox, 2010), thus intervals for comparisons are more likely to include zero (Gelman et al., 2012). A more traditional approach is to use the Bonferroni adjustment. However, this method is known to be a very conservative approach for controlling Type 1 errors, and the improved precision of the MLM approach is vastly superior to Bonferroni-adjusted intervals, which can drastically reduce power, resulting in Type 2 errors (Gelman et al., 2012). Regardless of our confidence in the MLM method, for the interested reader, we note the two contrasts in Table 3 that would not be statistically significant if the very conservative Bonferroni adjustment were applied to the already shrunk MLM coefficient estimates.

Model 1 explained 11.20% of the variance in the data. The linear effect of relational complexity was a statistically significant predictor of the log-odds of scoring correctly in the expected direction, RC: β_10_ = −0.51 (*SE* = 0.04, *z* = −14.22, *p* < .001). As expected, the interference condition (6 distractors) had significantly lower log-odds of success than the average of the baseline and facilitation conditions (0 and 12 distractors; HighD: β_30_ = −0.08, *SE* = 0.04, *z* = −1.96, *p* = .050). Additionally, as expected, the baseline condition (0 distractors) had significantly lower log-odds of success than the facilitation condition (12 distractors; LowD: β_40_ = −0.12, *SE* = 0.05, *z* = −2.70, *p* = .007). The relational complexity effect was not moderated by the level of distraction. Neither of the interaction coefficients were statistically significant (RC × HighD: β_50_ = 0.04, *SE* = 0.05, *z* = 0.73, *p* = .467; RC × LowD: β_60_ = 0.11, *SE* = 0.06, *z* = 1.95, *p* = .052).

Model 2 tested how relational complexity and levels of string preservation impact RMT performance (Table 3). Due to the near-singularity issue caused by the two string preservation contrasts, Model 2 estimated the random effect of the intercept and the linear contrast of relational complexity and explained 9.70% of the variance in the data. The linear and quadratic trends of relational complexity were consistent with Model 1. In regard to the preservation contrast, contrary to expectations, the average of 3- and 6-strings preserved trials had significantly lower log-odds chance of scoring RMT items correct than0-string preserved trials (PreserveC: β_30_ = 0.06, *SE* = 0.03, *z* = 2.12, *p* = .034). The log-odds chance of success did not significantly differ between 3-string preserved trials and 6-string preserved trials (PreserveL: β_40_ = 0.004, *SE* = 0.04, *z* = 0.08, *p* = .937). None of the interaction coefficients was significant (RC × PreserveC: β_50_ = 0.02, *SE* = 0.04, *z* = 0.67, *p* = .503; RC × PreserveL: β_60_ = −0.03, *SE* = 0.06, *z* = −0.50, *p* = .617).

RMT Association with Gf

Model 3 included Gf as a moderator in Model 1 and explained 11.20% of the variance in the data. Gf was a significant predictor of RMT performance in the direction expected (Gf: β_01_ = 0.27, *SE* = 0.05, *z* = 5.74, *p* < .001), none of the cross-level interactions were statistically significant (Gf × RC: β_11_ = −0.01, *SE* = 0.05, *z* = −0.25, *p* = .806; Gf × HighD β_31_ = 0.01, *SE* = 0.05, *z* = 0.19, *p* = .848; Gf × LowD β_41_ = −0.06, *SE* = 0.06, z = −0.91, *p* = .365). Model 3 explained less than .10% of variance in RMT over and above Model 1, though this was still statistically significant (χ^2^(4) = 30.76, *p* < .001). This is likely due to the significant main effect of Gf.

Model 4 included Gf as a moderator in Model 2 and explained 9.7% of the variance in the RMT data. Similarly to Model 3, Gf was a positive significant predictor of RMT performance (Gf: β_01_ = 0.22, *SE* = 0.05, *z* = 4.76, *p* < .001), and none of the cross-level interactions were statistically significant (Gf × RC: β_11_ = −0.05, *SE* = 0.04, *z* = −1.33, *p* = .187; Gf × PreserveC: β_31_ = 0.05, *SE* = 0.04, *z* = 1.26, *p* = .207; Gf × PreserveL: β_41_ = 0.06, *SE* = 0.06, *z* = 0.89, *p* = .371). Model 4 explained less than .10% variance in RMT performance over and above Model 2, though this was still statistically significant (χ^2^(4) = 28.80, *p* < .001). Again, this is likely due to the main effect of Gf significantly predicting the RMT performance.

#### 3.3.3. Simple-Composite Scores

For the simple-composite scores, we use a more traditional approach (e.g., as used by Bateman et al., 2019). The goal here is to quantify the incremental variance in Gf that these simple-composites account for. Incremental prediction at each step can be interpreted as the additional demand on Gf over and above the condition levels already in the model.

RMT Component 1 (Relational Integration and Visual Interference)

*Relational Integration:* The first regression model consisted only of the “same” composite, which significantly explained 6.65% of the variance in Gf (*F*(1, 144) = 11.03, *p* < .001). Adding RMT “ascending” composite into the model together explained 12.79% of variance in Gf (Δ*R*^2^ = .061, *F*(1, 143) = 11.92, *p* < .001). Adding RMT “different” composite into the model together explained 16.35% of variance in Gf (Δ*R*^2^ = .036, *F*(1, 142) = 7.10, *p* = .009). In the final model, each simple-composite explained a small but significant unique proportion of variance in Gf (same: sr2 = .03, *p* = .032; ascending: sr2 = .03, *p* = .028; different: *sr*^2^ = .04, *p* = .015).

*Attentional Control—Inhibition:* For visual interference, we regressed composite measures from RMT Baseline (0 distractors), Interference (6 distractors), and Facilitation (12 distractors), averaged over the same, ascending, and different trials, sequentially into an OLS regression model to predict Gf. The first model, which consisted only of the Baseline composite, significantly explained 9.98% of the variance in Gf (*F*(1, 144) = 17.08, *p* < .001). Adding RMT Interference composite into the model together explained 18.387 of variance in Gf (Δ*R*^2^ = .059, *F*(1, 143) = 11.18, *p* < .001). However, adding the RMT Facilitation composite into the model did not significantly explain more variance in Gf (Δ*R*^2^ = .009, *F*(1, 142) = 2.62, *p* = .119). In the final model, only the RMT Interference composite (6 distractors) uniquely explained Gf variance (*sr*^2^ = .03, *p* = .036).

RMT Component 2 (Relational Integration and String Preservation)

*Relational Integration:* The first model, which only consisted of the “same” composite measure, significantly explained 11.41% of variance in Gf (*F*(1, 144) = 19.68, *p* < .001). Adding RMT “ascending” composite into the model together explained 13.81% of variance in Gf (Δ*R*^2^ = .026, *F*(1, 143) = 5.08, *p* = .026). However, adding RMT “different” composite into the model did not significantly explain more variance in Gf (Δ*R*^2^ = .005, *F*(1, 142) = 1.82, *p* = .179). In the final model, only the same composite uniquely explained Gf variance (*sr*^2^ = .06, *p* = .004).

*Attentional Control—Scanning:* For string preservation, the study regressed composite measures from the RMT condition with 6, 3, and 0 strings preserved (averaged over the same, ascending, and different trials), sequentially into an OLS regression model to predict Gf. The first model, which consisted only of the 6-string preserved composite, significantly explained 4.52% of variance in Gf (*F*(1, 144) = 7.86, *p* = .006). Adding the 3-string preserved composite into the model together explained 9.74% of variance in Gf (Δ*R*^2^ = .052, *F*(1, 143) = 9.91, *p* = .002). Adding the 0-string preserved composite into the model together explained 15.64% of variance in Gf (Δ*R*^2^ = .060, *F*(1, 142) = 11.08, *p* = .001). In the final model, only the RMT 0-string preserved composite uniquely explained Gf variance (*sr*^2^ = .06, *p* = .002).

## 4. Discussion

The current study manipulated relational integration and attentional control demand in the RMT to understand the cognitive processes underlying Gf. The study adjusted the RMT response time for lower relational complexity conditions to minimise the ceiling effect, improved on the string preservation manipulation in Bateman et al. (2019), and compared the two analytic scoring methods, latent-contrasts and simple-composites. Overall, all but one RMT manipulations elicited variation in RMT performance in the predicted direction. However, conflicting results were found between simple-composites and latent-contrasts when examining the relationship between RMT performance and Gf.

### 4.1. RMT Performance (Hypothesis H1, H3, H4, and H6)

As the relational integration demand increases, RMT becomes more difficult, manifesting as having lower accuracy (H1). This supports the relational complexity theory and is consistent with previous RMT findings (i.e., Bateman et al., 2019; Chuderski, 2014; Zhan & Birney, 2023).

For visual interference manipulations of inhibition (H3), the distraction condition (RMT items with 6 distractors) had significantly poorer RMT performance than the average of baseline (0 distractors) and facilitation (12 distractors) conditions. This supports the arguments of Bateman et al. (2019) and Zhan and Birney (2023) that participants need to inhibit salient distractors when identifying matches in the RMT, which aligns with Attentional Control Theory. Further, the study also found that the facilitation condition (12 distractors) has significantly better performance than the baseline (H4). This aligns with the hypothesis of Bateman et al. (2019) that introducing too many distractors could cue participants to the target location, reducing the attentional control demand. Importantly, the current study adjusted the RMT response time to reduce the ceiling effect observed in Zhan and Birney (2023). This adjustment in Study 2 successfully elicited a decrease in mean RMT accuracy and an increase in SD in both the “same” and “ascending” conditions compared to Zhan and Birney (2023), suggesting a relief of the ceiling effect for lower relational complexity conditions.

For the visual scanning manipulation, RMT items with three- and six-strings preserved showed poorer RMT performance than those with zero-strings preserved (H6). This result contradicts our predictions based on Attentional Control Theory. One possible explanation might relate to the disengagement processes. As participants were not informed that strings could be retained after a trial finished, the disengagement processes might be instantiated to eliminate the current representation of strings in WM, at least for some participants. This could help prevent outdated information from interfering with future processing (Shipstead et al., 2016). However, when strings are preserved, these removal processes ultimately discard useful representations. This may confuse the WM system, leading to reduced WM efficiency.

Nonetheless, an alternative account is that the string-preservation manipulation might not be valid for distinguishing between attentional control and relational integration in RMT. That is, it may be the case that when strings are preserved, not only are the demands of attentional control reduced, but also the binding demands (the binding between the target digits of RMT and spatial coordinates in WM), which are an essential process of relational integration. To explain the current result, one may also interpret it as a disruption of the binding and unbinding processes, rather than the disruption of the disengagement processes. Due to this failure to tease apart relational integration and attentional control, we decided to exclude it from our interpretation when analysing the relationship between RMT and Gf.

### 4.2. RMT and Gf (Hypotheses H2, H5, and H7)

When examining the relationship between RMT performance and Gf, contradictory findings appeared depending on the analytic approach used. When using latent-contrast scores via MLM, only Gf significantly predicted participants’ overall RMT accuracy as a main effect. Although this aligns with previous findings that RMT performance is highly correlated with Gf (Chuderski, 2014; Bateman et al., 2019), Gf did not significantly moderate the cost of increasing relational integration and attentional control demand in the RMT in these models. This seems to contradict most previous empirical findings on both relational integration and attentional control.

When applying OLS regression to analyse simple-composites from the same data, all previous key findings except for the string preservation manipulation were replicated. When a higher relational complexity condition is entered into the model, it significantly better predicts Gf over and above the lower relational complexity condition(s). For visual interference, the pattern remains the same as relational complexity. From OLS regression models, this evidence suggests that relational integration and attentional control are processes associated with Gf.

This discrepancy of results may be explained when comparing the two types of models. Specifically, when using simple-composites in an OLS regression, which lacks the nuanced variance decomposition to differentiate between attentional control and relational integration in RMT, both significantly predicted Gf. However, when using latent-contrasts and MLM, which can statistically separate attentional control and relational integration, none of them alone seems to be related to Gf. Importantly, in MLM, Gf also significantly and positively predicted the grand mean of RMT performance (intercept) averaged over both attentional control and relational integration manipulations. Therefore, to integrate the findings from both analyses, relational integration and attentional control processes seem to be crucial to fluid intelligence when operationalised together but not alone.

It is important to note that there are studies that utilised MLM with a similar experimental-individual difference approach and found evidence for relational integration processes related to Gf. Bateman and Birney (2019) used a similar experimental individual differences approach with latent contrasts using MLM and found support for relational integration using the arithmetic chain task. They extracted the covariance between their complex span task and RPM measures to represent the commonality between WM and Gf. They found that the commonality between WM and Gf significantly moderated the cost of increasing relational integration demand. Specifically, participants who scored higher on the WM–Gf composite decreased less in task performance as relational integration demand increased than participants who scored lower on the WM–Gf composite. Similarly, by manipulating the relational integration demand in the Latin Square Task, Birney and Zhan (2023) found that people with higher Gf incurred less cost on task performance when relational integration demand increased than people with lower Gf (a significant moderation). Meanwhile, recent attentional control studies developed improved measures to tackle the reliability issues of traditional assessments (Draheim et al., 2021, 2024). They found that the attentional control factor extracted from the improved tests fully explained the WM–Gf relationship.

Although the above cited studies improved their approaches by either adopting a more suitable statistical approach or improving the test measures, they did not simultaneously control for both attentional control and relational integration processes, as our study did. Specifically, neither Bateman and Birney (2019) nor Birney and Zhan (2023) controlled for attentional control processes when investigating the relationship between relational integration and Gf. Similarly, when Draheim et al. (2021) extracted the improved attentional control factor from the improved reliability measures, they did not control for relational integration processes. From this perspective, the results of these studies also align with our current finding that relational integration and attentional control processes are crucial to fluid intelligence together but not alone.

### 4.3. Alternative Explanations, Limitations, and Future Research

As discussed above, our proposed hypothesis may explain the current inconsistencies, both within this study and in comparison to others in the literature. However, alternative explanations still exist. Birney and Zhan (2023) manipulated relational complexity in the Latin Square task and induced a 30–40% decrease in task accuracy. However, with the RMT in the current study, we only observed a 10–20% decrease across levels of relational complexity. Furthermore, the attentional control manipulation in the current study resulted in only a 5% decrease in task performance. Therefore, the effects are small, and it is possible that our manipulations were still not distinct enough to observe the interaction with Gf in MLM. To address this alternative explanation, future studies might manipulate the attentional control demand in other tasks where stronger relational integration effects have been observed, such as the Latin Square task, and use a latent-contrast approach to investigate how the unique latent relational integration and attentional control variables are associated with Gf.

Another limitation of the current study comes from the string preservation manipulation. Contrary to the predictions from both relational integration and attention control perspectives, RMT performance was worse when strings were preserved across trials. One may argue that Gf did not significantly moderate the difference between levels of string preservation, because of this. It is unclear how to interpret the results from the string preservation manipulation. Arguably, a stronger, more elaborated theoretical explanation than that provided by Bateman et al. (2019) is needed to understand the cognitive processes involved in scanning operationalised as string preservation in the RMT.

Finally, an important future direction involves identifying and contrasting different relational integration processes (rather than treating them as one), such as content-context binding and the integration of bindings (e.g., Oberauer, 2021) and investigating how they are related to Gf.

## 5. Conclusions

The current study aimed to investigate two common cognitive process accounts associated with Gf. The adjusted RMT response time window addressed the ceiling effect observed in previous studies, which aimed to amplify the impact of attentional control on RMT trials of lower levels of relational complexity. By using the combined experimental-individual differences paradigm and operationalising performance with latent-contrast scores, the study addresses a reliability challenge in attentional control measures and a validity challenge in the use of simple-composite scores. We also improved the string preservation manipulation, thereby rectifying Bateman et al.’s (2019) prior ambiguous operationalisation of attentional control. However, the manipulation still failed to provide clear insight into the association between scanning and Gf. Based on the latent-contrast approach, our results suggest that both the attentional control and relational integration accounts of Gf alone may be incomplete. On the other hand, when relational integration and attentional control are taken together in simple-composite scores, both sets of processes seem to be important in understanding individual differences in Gf. The discrepancy between these different analytic approaches and outcomes raises important conceptual challenges in the scoring and testing of cognitive process accounts. It highlights a need to more thoroughly understand how insights gleaned from experimental studies are influenced by operationalisation decisions.

## Figures and Tables

**Table 1:** Response window conditions for different criteria used in study 1.

Relational Complexity	Upper Quartile Response Time	Median Response Time	10% Timeout Rate
Same	3.80 s	2.65 s	4.05 s
Ascending	4.41 s	3.47 s	4.72 s
Different	4.71 s	3.62 s	5.00 s

Note: Participants in Study 1 were randomly allocated into one of these three groups (upper quartile, median, and 10% timeout rate).

**Table 2:** Descriptive statistics of simple composites from each component and correlation with Gf.

	Descriptive Statistics		Correlation
	*M*	*SD*	Gf
RMT Grand Total	.70	.08	.43 **
RMT Component 1			
RMT Same	.81	.11	.27 *
RMT Ascending	.67	.12	.32 **
RMT Different	.62	.14	.31 **
RMT 12 Distractors	.71	.11	.33 **
RMT 0 Distractor	.69	.10	.39 **
RMT 6 Distractors	.69	.10	.37 **
RMT Component 2			
RMT Same	.81	.09	.35 **
RMT Ascending	.66	.11	.28 **
RMT Different	.63	.11	.27 **
RMT 6 Preservation	.69	.10	.23 *
RMT 3 Preservation	.69	.09	.33 **
RMT 0 Preservation	.70	.08	.40 **
Gf Measures			
RPM	.54	.19	.67 **
Number Series	.57	.19	.70 **
GLST	.56	.18	.74 **

Note: The means represent the average accuracy. For example, M = 1.0 represents an average accuracy of 100%. The correlations are Pearson correlation. All correlations with Gf are significant (* *p* < .01, ** *p* < .001).

**Table 3:** Fixed and random effect estimates of planned contrasts in MLM.

			Fixed Effects				Random Effects
Predictors	Model	Log-Odds	*SE*	*z*	*CI*	*p*	tau
RC (linear contrast; H1)	1	−0.512	0.036	−14.22	−0.583, −0.442	**<.001**	0.17
RC.quad (quadratic contrast)	1	0.287	0.038	7.58	0.213, 0.361	**<.001**	
HighD (high distraction contrast; H3)	1	−0.075	0.038	−1.96	−0.151, −0.000	.050 *	
LowD (facilitation vs. baseline; H4)	1	−0.121	0.045	−2.70	−0.209, −0.033	**.007**	
RC × HighD	1	0.035	0.048	0.73	−0.059, 0.130	.467	
RC × LowD	1	0.110	0.056	1.95	−0.001, 0.220	.052	
RC (H1)	2	−0.490	0.027	−17.83	−0.544, −0.436	**<.001**	0.051
PreserveC (preserve cost; H6)	2	0.059	0.028	2.12	0.004, 0.113	.034 *	
PreserveL (preserve levels; H6)	2	0.004	0.044	0.08	−0.083, 0.090	.937	
RC × PreserveC	2	0.023	0.035	0.67	−0.045, 0.092	.503	
RC × PreserveL	2	−0.028	0.055	−0.50	−0.136, 0.081	.617	
Gf	3	0.271	0.047	5.74	0.178, 0.363	**<.001**	
Gf × RC (H2)	3	−0.013	0.051	−0.25	−0.113, 0.088	.806	
Gf × HighD (H5)	3	0.010	0.054	0.19	−0.096, 0.116	.848	
Gf × LowD (H5)	3	−0.057	0.063	−0.91	−0.181, 0.066	.365	
Gf	4	0.219	0.046	4.76	0.129, 0.309	**<.001**	
Gf × RC (H2)	4	−0.047	0.036	−1.32	−0.118, 0.023	.187	
Gf × PreserveC (H7)	4	0.049	0.039	1.26	−0.027, 0.126	.207	
Gf × PreserveL (H7)	4	0.056	0.062	0.89	−0.066, 0.178	.371	

Note: SE = standard error. CI = 95% confidence interval. H1 to H7 refer to hypotheses 1–7 stated in the Introduction. **Contrasts Effect-Coding**: RC = (Same = −1; Ascending = 0; Different = 1); RC.quad = (Same = 1/3; Ascending = −2/3; Different = 1/3); HighD = (Facilitation (12) = −1/3; Baseline (0) = −1/3; Interference (6) = 2/3); LowD = (Facilitation (12) = −0.5; Baseline (0) = 0.5; Interference (6) = 0); PreserveC = (6 Strings = −1/3; 3 Strings = −1/3; 0 Strings = 2/3); PreserveL = (6 Strings = −0.5; 3 Strings = 0.5; 0 Strings = 0). **Model 1**: glmer(RMT ~ 1 + RC × HighD + RC × LowD + RC.quad + (1 + RC | subject). **Model 2**: glmer(RMT ~ 1 + RC × PreserveC + RC × PreserveL + RC.quad + (1 + RC | subject). **Model 3**: glmer(RMT ~ 1 + RC × HighD + RC × LowD + rc.quad + RC × Gf + HighD × Gf + LowD × Gf + (1 + RC | subject). **Model 4**: glmer(RMT ~ 1 + RC × PreserveC + RC × PreserveL + rc.quad + RC × Gf + PreserveC × Gf + PreserveL × Gf + (1 + RC | subject). The *p*-values in bold are statistically significant at *p* ≤ .05. * These effects would not be statistically significant under the very conservative Bonferroni adjustment (see note in text).

## Data Availability

The original data presented in the study are openly available in OSF at https://osf.io/25967/overview?view_only=ec70bfea389b49c997e87b5e2ee911ac (accessed on 12 December 2025).

## Note

1 Re-analysis of Bateman et al. (2019) by Zhan and Birney (2023) suggested that the “same” rule should be classified as a unary relation for the reasons outlined here and in Figure 1, and not binary as Bateman et al. had originally proposed.
